# Efficacy of Polyunsaturated Fatty Acids (PUFAs) on Impulsive Behaviours and Aggressiveness in Psychiatric Disorders

**DOI:** 10.3390/ijms22020620

**Published:** 2021-01-09

**Authors:** Silvio Bellino, Paola Bozzatello, Cristina Badino, Emanuela Mantelli, Paola Rocca

**Affiliations:** Center for Personality Disorders, Psychiatric Clinic, Department of Neuroscience, University of Turin, Via Cherasco 11, 10126 Turin, Italy; paola.bozzatello@unito.it (P.B.); cristina.badino@unito.it (C.B.); emanuela.mantelli@unito.it (E.M.); paola.rocca@unito.it (P.R.)

**Keywords:** long-chain n-3 polyunsaturated fatty acids, long-chain n-6 polyunsaturated fatty acids, PUFAs, impulsive behaviours, aggressiveness, psychiatric disorders

## Abstract

It is the focus of increasing interest to investigate the effects of long-chain n-3 and long-chain n-6 polyunsaturated fatty acids (LC n-3 PUFAs; LC n-6 PUFAs) on psychiatric symptoms in a transdiagnostic perspective. There is some evidence that low levels of LC n-3 PUFAs and a higher ratio of LC n-6 to LC n-3 PUFAs in plasma and blood cells are associated with aggressive and impulsive behaviours. Therefore, implementation of LC n-3 PUFAs may produce positive effects on hostility, aggression, and impulsivity in both psychiatric and non-psychiatric samples across different stages of life. A possible mechanism of action of LC n-3 PUFAs in conditions characterized by a high level of impulsivity and aggression is due to the effect of these compounds on the serotonin system and membrane stability. Studies that evaluated the effects of LC n-3 PUFAs on impulsivity and aggressiveness indicated that addition of rather low doses of these agents to antipsychotic treatment might reduce agitation and violent behaviours in psychosis, attention deficit hyperactivity disorder, personality disorders, and impulsive control and conduct disorders. The present review is aimed at examining and discussing available data from recent trials on this topic.

## 1. Introduction

In the last two decades, there has been an increasing interest among investigators in the role of PUFAs in human health [1]. These agents cannot be synthesized by the human body but are obtained from diet and metabolic conversion [2]. The main long-chain n-3 PUFAs (LC n-3 PUFAs) include alpha linolenic acid (ALA), eicosapentaenoic acid (EPA), and docosahexaenoic acid (DHA), while the long-chain n-6 PUFAs (LC n-6 PUFAs) primarily involve gamma-linolenic acid (GLA) and arachidonic acid (AA).

Among LC n-3 PUFAs properties, they produce a protective effect on the inflammatory and cardiovascular systems, improving the lipid and lipoprotein profile and the endothelial function [3], and influence the downregulation of leukocyte cells and the concentrations of proinflammatory biomarkers related to atherosclerosis, such as chemokines and cytokines [4].

The role of LC n-3 PUFAs, essential compounds of the neuronal cell membranes, is now recognized in several brain functions. Some studies report that poor intake of EPA and DHA during the neurodevelopmental process produces consequences in terms of reduction of serotonin synthesis, storage, and release; impaired receptor function [5]; and deficit in neurogenesis, dendritic arborization, synaptogenesis, selective pruning, and myelination [6]. On the other hand, their supplementation can contribute to the prevention of the onset of cognitive disorders by the reduction of the neurotoxicity typical of neurodegenerative disorders [7,8]. Moreover, there is some evidence that higher levels of LC n-3 PUFAs are associated with the improvement of stress coping in animal models [9].

Nutritional intervention trials on omega-3 fatty acids have been performed to test the effects of the intake of two important LC n-3 PUFAs, EPA and DHA. As these agents are not produced de novo by the body, but are obtained from their conversion from ALA or through dietary intake, experimental approaches are based on dietary supplementation. There is a growing interest in the nascent field of “nutritional psychiatry” and the potential for nutritional interventions to help improve mental health problems [10]. In particular, the discovery of low levels of EPA and DHA in the central nervous system of subjects with impulsive, aggressive, self-harm, and parasuicidal behaviours has encouraged investigators to conduct trials on LC n-3 fatty acids’ implementation [1,11,12,13,14,15,16]. These studies have been partly stimulated by findings from longitudinal studies showing that poor nutrition in infancy is associated with aggressive and antisocial behaviours [17], and even maternal diet poor of LC n-3 PUFAs during pregnancy is a predisposing factor of antisocial personality disorder in offspring [18].

The association between deficit of LC n-3 PUFAs and aggressive and impulsive behaviours has been observed in children and adolescents with conduct disorders and attention deficit hyperactivity disorder (ADHD) and in adults with schizophrenia, bipolar disorder, and personality disorders. Not only a lower plasma level of LC n-3 PUFAs but also an unbalanced LC n-6/LC n-3 PUFAs ratio was observed in psychiatric patients who expressed impulsive dyscontrol as the main symptom cluster, such as impulsive gamblers [19] and cocaine abusers [20].

The relationship between decreased LC n-3 PUFA availability and psychiatric conditions with high levels of impulsivity could be partly explained by the effect of these compounds on the serotonin system that is implicated in inhibiting impulsive aggression [21]. Depleting brain serotonin levels in normal individuals results in increased antisocial behaviours, uncontrolled aggression, and outbursts of anger [22]. On the opposite side, the stimulation of EPA on serotonin release and of DHA on membrane-embedded serotonin receptor accessibility produces a positive effect on cognitive functioning, propensity for prosocial behaviours, and control of impulsive reactions [23,24,25].

It is the focus of increasing interest to investigate the effects of polyunsaturated fatty acids (PUFAs) on dimensions of impulsivity and aggression in a transdiagnostic perspective [26,27]. The present review is aimed at examining available evidence on the effects of LC n-3 and n-6 PUFAs on these psychopathological dimensions throughout patients with psychotic disorders, personality disorders, attention-deficit hyperactivity disorder, and impulse control and conduct disorders.

## 2. Efficacy of PUFAs on Impulsive Behaviours and Aggressiveness in Schizophrenia

Schizophrenia is a mental disorder characterized by delusions, hallucinations, disorganized symptoms, apathy, abulia, anhedonia, social withdrawal, and cognitive deficits. Aggression and agitation in patients with schizophrenia and related psychoses are acute symptoms that constitute one of the main causes for admission to psychiatric hospitals [28].

Violent behaviours in schizophrenia can be determined by several conditions, such as the presence of positive psychotic symptoms, comorbidity with personality disorders, and a high level of impulsivity. Other factors that can contribute to aggressive conducts are neurological abnormalities, concomitant substance use, and poor medication adherence [29]. In recent years, some studies have investigated in patients with different stages of psychosis the potential correlation between impulsive–aggressive behaviours and low plasma levels of LC n-3 PUFAs [30,31]. For example, a study performed in patients with acute drug-free schizophrenia suggested that the concentration of fatty acids in red blood cells was inversely related with the hostility score in the Positive and Negative Syndrome Scale (PANSS) [32].

Current treatment of impulsive and aggressive behaviours in schizophrenia mainly relies on antipsychotics. In particular, the role of clozapine in reducing hostility and aggression in patients with psychosis is generally recognized [33,34]. On the basis of the relationship between low LC n-3 PUFA plasma levels and impulsivity and aggressiveness in schizophrenia, some trials were performed with the intent to evaluate the benefits of supplementation of EPA and DHA in the treatment of this disorder, in monotherapy or more frequently in association with antipsychotic drugs.

The majority of clinical trials performed in schizophrenia were aimed at testing the efficacy of these agents in reducing the rate of progression to psychosis in high-risk subjects [35,36] or in improving positive and negative symptoms of patients with stable schizophrenia [37,38,39].

A limited number of studies have specifically evaluated the effects of EPA and DHA in the domains of impulsivity and aggressiveness in patients with schizophrenia.

Three clinical trials and one pilot study analysed the improvement of impulsiveness, aggressiveness, and hostility in patients with schizophrenia treated with LC n-3 PUFAs. One trial was conducted in adolescents at high risk of developing psychosis [35], while three studies were performed in patients with stable schizophrenia [40,41,42]. Trial duration ranged between 8 and 12 weeks; treatment doses of EPA and DHA ranged, respectively, between 0.54 and 1.2 g/day and between 0.36 and 0.6 g/day. In two studies [41,42], LC n-3 PUFAs were administered in addition to antipsychotics. In one study [35], they were used in monotherapy or in addition to antidepressants or anxiolytics. A study by Légaré et al. [40] focused on the treatment of resistant patients without specifying the use of previous medications (only benzodiazepine was mentioned). The evaluation instruments used to measure poor impulse control and aggressiveness/hostility were the PANSS and the Modified Overt Aggression Scale (MOAS).

This pilot study published by Légaré et al. [40] was conducted on 12 violent male inpatients with chronic schizophrenia. Data suggested that nutritional supplement with EPA and DHA may facilitate the reduction of agitation and global psychopathology among these patients. These promising results were confirmed by Qiao et al. [41] in a larger sample of 50 patients (30 males and 20 females) with stable schizophrenia and aggressiveness (MOAS score higher than 4) who were randomly assigned to receive either fish oil (*n* = 28) or placebo (*n* = 22) in a double-blind trial. MOAS scores declined more in the LC n-3 PUFAs group than in the placebo group at the end of the study period. Therefore, the authors concluded that patients with schizophrenia and aggressive/violent behaviours treated with PUFAs showed a mitigation of their abnormal conducts. In this study was not found a relationship between baseline plasma level of EPA and DHA and level of aggression (MOAS scores) in the enrolled subjects.

In a study conducted by Amminger et al. [35], 15 adolescents (14 were female, 1 male) were evaluated. They received LC n-3 PUFAs (*n* = 8) or placebo (*n* = 7) in a random way. The participants who were treated with EPA and DHA supplementation experienced a greater improvement in impulsivity than those in the placebo group. In addition, suspiciousness, inner tension, emotional withdrawal, and anxiety showed a significant decrease during the consumption of LC n-3PUFAs.

On the opposite side, a study conducted by Qiao et al. [42] reported negative results. In fact, patients treated with EPA and DHA did not show a significant improvement of hostility and aggression after treatment. This double-blind, placebo-controlled trial was conducted in 67 acute hospitalized patients with schizophrenia (35 males and 32 females) who showed violent and aggressive behaviours. They were randomly assigned to a group with supplementation of LC n-3 PUFAs (*n* = 32) or a placebo group (*n* = 35) in addition to antipsychotics. A possible explanation of the negative results could be that in this study, the patients received a rather low concentration of EPA and DHA. Moreover, the authors administered vitamin E as placebo treatment, while in other studies (e.g. Légaré et al. [40]), vitamin E was supplemented together with LC n-3 PUFAs.

Although there are some encouraging data about the relationship between PUFAs and impulsive and aggressive behaviours, findings remain inconclusive because of small sample sizes and scarcity of trials that specifically investigate these symptom domains.

Results of randomized controlled trials (RCTs) are displayed in detail in Table 1. 

## 3. Efficacy of PUFAs on Impulsive Behaviours and Aggressiveness in Personality Disorders

Personality disorders are enduring maladaptive patterns of behaviours, cognition, and inner experience exhibited across many contexts and deviating from those accepted by the individual’s culture. These patterns develop early, are inflexible, and are associated with significant distress or disability. Among personality disorders, the effects of LC n-3 PUFA supplementation have been investigated in borderline personality disorder (BPD), in which impulsivity, inappropriate and intense anger, and aggressiveness constitute the core characteristics of the disease. Patients with BPD act on the spur of the moment without consideration of the consequences, have difficulty establishing a plan, have a sense of urgency, present self-harm behaviours, and engage in potentially self-damaging activities. Moreover, these patients have a certain degree of persistent hostility, frequent outburst of anger, and irritability in response to minor slights and insults. Around 0.5% of BPD patients in the community manifest externalized aggression in the form of interpersonal violence [43]. BPD shares the traits of impulsivity, irritability, anger, and propensity to engage in physical violence with antisocial personality disorder (ASPD) [43].

With regard to neurobiological basis, impulsive aggression in BPD has been defined as an unbalance between the “top–down” control maintained by frontal cortices [44,45,46,47] and the “bottom–up drives” provided by limbic regions [48]. The hippocampus may play an important role in the regulation of aggressive behaviours [49], and an inverse correlation between hippocampal volume and aggressive traits has been found in BPD patients [50].

The effects of supplementation of LC n-3 PUFAs in BPD consist in the improvement of core symptoms of BPD, like depression [51,52,53], self-harm behaviours [52,53,54,55], and stress reactivity [52].

In particular, four RCTs [35,51,55,56] focused on the effects of EPA and DHA on the dimensions of impulsivity, aggression, and anger in BPD. Three trials reported positive results of LC n-3 PUFA supplementation in reducing aggressiveness [51,55,56], while two studies showed an improvement of impulsive behaviours [35,55]. On the other hand, one follow-up study [56] did not obtain any evidence of long-lasting effects of LC n-3 PUFAs on impulsive behavioural dyscontrol.

Three studies [51,55,56] were performed on patients with borderline personality disorder, while one study [35] involved individuals at ultra-high risk (UHR) of developing psychosis, including 15 patients who had a diagnosis of BPD.

One study tested the effect of EPA alone [51], while the remaining ones studied the effects of the combination of EPA and DHA. Amminger et al. [35] administrated also vitamin E (0.76 g/die) together with EPA and DHA. Daily PUFA doses ranged from 0.7 to 1.2 g for EPA and from 0.4 to 0.6 g for DHA. RCTs’ duration ranged from 8 to 12 weeks [35,51,55]. Only one 24-week follow-up study was published [56].

The evaluation instrument used to assess aggressiveness was the same across trials (Modified Overt Aggression Scale (MOAS)). Bellino et al. [55] and Bozzatello et al. [56] studied impulsivity with the Barratt Impulsiveness Scale and used also the items “outbursts of anger” and “impulsivity” of the Borderline Personality Disorder Severity Index (BPDSI) to investigate, respectively, aggressiveness and impulsivity. In a study conducted by Amminger et al. [35], impulsive behaviours were assessed with the item “poor impulse control” of the Positive and Negative Syndrome Scale (PANSS).

The first study, published by Zanarini and Frankenburg [51], studied levels of aggressiveness and depression in a group of 30 BPD women with a mean age of 26.3 years, who were treated for 8 weeks with ethyl-eicosapentaenoic acid (E-EPA) (*n* = 20) or placebo (*n* = 10), in the absence of other psychotropic medications. Results led to the conclusion that the E-EPA group experienced a significantly greater decrease in overall aggression compared with the placebo group.

Amminger et al. [35] assessed a group of 15 adolescents with BPD who also met the criteria for UHR of psychosis. The sample was composed of 14 females and 1 male with a mean age of 16.2 years, randomized to receive either LC n-3 PUFAs with vitamin E (*n* = 8 treated with EPA + DHA) or placebo (*n* = 7) for a period of 12 weeks. The study investigated several symptoms, such as suspiciousness or persecution, tension, emotional withdrawal, anxiety, and poor impulse control, together with levels of social, occupational, and psychological functioning. In particular, impulsivity showed a significantly greater improvement in the group treated with LC n-3 PUFAs.

Two studies [55,56] compared for 12 weeks combined therapy with EPA plus DHA and valproic acid (*n* = 23) with single therapy with valproic acid (*n* = 20) in 43 patients with BPD aged between 18 and 50 years. The findings showed a more pronounced reduction in the severity of self-rated and clinician-rated impulsive behavioural dyscontrol, outbursts of anger, and self-mutilating conduct in the group of patients treated with the association of valproate and LC n-3 PUFAs. In a follow-up study [56], the positive effect on impulsivity was lost, while the beneficial effect was maintained on outbursts of anger, which is a symptom domain often linked to impulsive and aggressive dyscontrol.

Therefore, in borderline personality disorder, supplementation of LC n-3 PUFAs achieved positive effects on reducing impulsivity, self-injuries, and anger, which are core symptoms of this pervasive mental illness. The results in these dimensions are encouraging, but evidence is still limited. If initial positive results were confirmed, LC n-3 PUFAs (EPA and DHA) would be a valid option for patients with personality disorders, together with standard treatment, such as antipsychotics and mood stabilizers [57,58]. Furthermore, findings suggest that n-3 PUFAs may also be an effective and well-tolerated treatment in adolescents with BPD who are at risk of developing psychosis.

Results of RCTs are displayed in detail in Table 2. 

## 4. Efficacy of PUFAs on Impulsive Behaviours and Aggressiveness in Impulse Control and Conduct Disorders

Disruptive, impulse-control, and conduct disorders are externalizing psychopathological disorders characterized by problems in self-control of emotion and behaviours [59]. They include poor impulse control, emotional dysregulation, and superficial affection. They also encompass antisocial behaviours with violation of social norms and rights of others through delinquency, property damage, aggressiveness, and rebellion against authority figures. These abnormal conducts represent a public health problem as they have a precocious onset in childhood or adolescence and are associated with substance abuse and violent and criminal behaviours in early stages of life [60,61].

### 4.1. Impulsive and Aggressive Behaviours in Subjects with No Diagnosis of Psychiatric Disorders

Even in the absence of a full diagnosis of psychiatric disorder, individuals with antisocial traits or violent behaviours showed a reduction in aggressive and impulsive conducts with the supplementation of dietary nutrients, including LC n-3 PUFAs [60,62,63,64]. Poor nutritional status at the age of 3 years has been found to predispose to externalizing behavioural problems throughout childhood and adolescence [17]. In particular, low plasma levels of total LC n-3 PUFAs and a trend towards a higher ratio of LC n-6 to n-3 PUFAs were observed in conditions with a high level of impulsivity and anger. For example, a lower plasmatic level of DHA was observed in habitually violent and impulsive male offenders compared with nonviolent individuals [65,66].

There is evidence of the effectiveness of LC n-3 PUFAs in reducing aggressive conducts in the non-psychiatric young population. For example, in a 12-week double-blind RCT conducted in UK adolescents aged 13–16 years, subjects from troubled backgrounds presented very low blood levels of DHA + EPA, and supplementation with these agents was useful to reduce the level of disruptive conducts, especially in adolescents with a high initial level of violations [67]. Similar results were observed in a sample of 8- to 16-year-old children who received EPA and DHA supplementation in the form of fruit juice drink during a period of 3 months. In this population, a statistically significant reduction in externalizing and internalizing behavioural problems was observed, with improvement continuing 6 months after treatment cessation [68]. LC n-3 PUFA supplementation was also tested in a large group of 324 schoolchildren with a mean age of 11.9 years, with positive results on early-onset antisocial and aggressive behaviours particularly in females, as well as in children with psychopathic-like traits [69].

### 4.2. Impulsive and Aggressive Behaviours in Subjects with a Diagnosis of Psychiatric Disorders

Concerning clinical samples, one observational study [70] and three RCTs [71,72,73] were performed in patients with high risk of externalizing conducts or patients specifically diagnosed with externalizing behavioural disorders.

In the observational study, 942 children with ages ranging between 6 and 12 years with behavioural problems were monitored for 3 months. Subjects who received a supplementation of EPA and DHA showed a significant improvement in externalizing conducts [70]. The three RCTs evaluated the effects of LC n-3 PUFAs on aggressive and antisocial behaviours. One was performed in children at high risk of externalizing conducts, the others in young individuals specifically diagnosed with externalizing behavioural disorders [71,72,73]. All the three studies obtained positive results about the efficacy of supplementation with LC n-3 PUFAs in reducing aggressive and antisocial behaviours. More in detail, the first trial was conducted on young adult prisoners showing antisocial behaviour [71], the second compared LC n-3 PUFAs with cognitive behaviour therapy [72], and the third was performed by the same authors to evaluate LC n-3 PUFAs versus social skill interventions [73]. All studies tested the effects of the combination of EPA and DHA with daily doses ranging from 0.08 to 0.6 g for EPA and from 0.04 to 0.4 g for DHA. RCTs’ duration ranged from 3 to 9 months [71,72,73]. The evaluation instruments used to assess aggressiveness and antisocial behaviour were the same in the two trials performed by the same research group [72,73] and were represented by the Reactive–Proactive Aggression Questionnaire (RPQ), the Child Behaviour Checklist (CBCL), the Youth Self-Report (YSR), the Antisocial Personality Screening Device (APSD), the Conduct and Oppositional Defiant Disorder Scales (CODDS), and the Aggression Questionnaire (AQ). On the other hand, Gesh et al. [71] used the Survey Anger Scales to assess outbursts of anger and aggression.

Gesh et al. [71] studied aggressive and antisocial behaviours in 172 young adult prisoners with externalizing disorder, comparing the rates of disciplinary offences before and after supplementation with EPA and DHA along a period from a minimum of 2 weeks to a maximum of 9 months. The average time spent on supplementation was the same for the two groups: 142 days for the placebo group (*n* = 90) and 143 days for the active group (*n* = 72). Compared with the baseline, the effect on prisoners treated with supplements of PUFAs for a minimum of 2 weeks was calculated to be a 35.1% reduction in violent offences.

Raine et al. [72] analysed a sample of 290 children at high risk of conduct disorders aged between 11 and 12 years, who were randomly assigned to a group receiving LC n-3 PUFAs (EPA + DHA) alone (*n* = 72) or to a group receiving cognitive behavioural therapy (CBT) alone (*n* = 73) or to a group receiving LC n-3 PUFAs plus CBT (*n* = 73) or to a group receiving placebo (*n* = 72). Children who received LC n-3 PUFA supplementation showed a significant reduction in externalizing behaviours compared with the control group after 3 months of treatment. Furthermore, in children who received a combination of LC n-3 PUFAs and CBT were observed lower levels of externalizing behaviour in comparison with both: those who received CBT alone and those who received placebo at 6 months of follow-up [72]. Similar encouraging findings were obtained from another study performed by the same research group [73] on a clinical sample of 282 children with externalizing behaviour aged between 7 and 16 years who were randomly treated for 6 months with LC n-3 PUFAs (*n* = 67), social skills training (*n* = 66), a combination of LC n-3 PUFAs and social skills (*n* = 75), and placebo (*n* = 74). The outcome measures were reactive aggression and antisocial behaviour. The results suggested that LC n-3 PUFA supplementation produced a significant long-lasting decline in child-reported reactive aggression at 12 months of follow-up.

Among other psychopathological conditions characterized by poor impulse control, special attention should be paid to gambling disorder (GD) and substance use disorders (SUDs).

GD is a “behavioural” addiction characterized by an uncontrollable urge to keep gambling despite negative consequences on the individual’s life. In these conditions, dopamine dysregulation in the orbitofrontal and anterior cingulate cortices may be partially responsible for the compulsive behaviour and impulsivity [74]. Moreover, gambling, as well as drugs or alcohol, stimulates the reward system of the brain, which is responsible for the addiction behaviours [75]. Both individuals with SUDs and subjects with gambling disorder show impairments in risky decision making and in reflection impulsivity in comparison with control subjects [76]. This process may be mediated by diminished top–down control of the prefrontal cortex over subcortical processes, promoting motivations to engage in addictive behaviour [77].

In patients with gambling disorder, and in particular in more impulsive ones, a higher percentage composition of EPA and a lower arachidonic acid (AA)/EPA ratio and AA/DHA ratio were found in membranes of red blood cells. On the other hand, EPA seems to have a beneficial role in modulating physical and psychological symptoms associated with craving in addiction conducts throughout the decrease of inflammatory cytokines’ toxicity [78].

To substantiate the hypothesis that a link exists between omega-3 fatty acid deficiency and aggression in addiction, some authors reported that in patients with cocaine abuse and aggressive behaviours, there were lower plasma levels of DHA and a significant increase in the ratio of LC n-6 to LC n-3 PUFAs [20,79]. On the opposite side, other investigators reported that low plasma levels of EPA, but not of DHA, were associated with a higher degree of aggression and impulsivity in patients with major depression and comorbid substance use disorder [80].

The efficacy of LC n-3 PUFAs on aggressive behaviours in substance use disorders was investigated in two studies conducted by Buydens-Branchey in 2008 [79,81]: an RCT and a follow-up study, which compared EPA and DHA with placebo. These trials tested the effects of a combination of EPA and DHA at high doses: 3 g of EPA and 3 g of DHA administrated for 3 months and during a follow-up period of further 3 months. The evaluation instrument used to assess aggressiveness was anger score obtained with the modified version of the Profile of Mood States (POMS). Twenty-four men with substance use disorder were randomized to receive LC n-3 PUFAs (*n* = 13) or placebo (*n* = 11). The findings suggested that treatment with LC n-3 PUFAs was superior to that with placebo in diminishing outbursts of anger. These changes were associated with an increase in plasma in both EPA and DHA, but higher EPA levels were more robustly correlated with a reduction in anxiety, while an increase in DHA was more significantly associated with a reduction in outbursts of anger/aggression [79]. These favourable results were confirmed during a period of 3 months following treatment discontinuation [81].

Recent research about supplementation of LC n-3 PUFAs in patients with impulsive and aggressive/antisocial behaviours suggests that the efficacy of a dietary intervention in both children and adults could be stronger for more impulsive forms of aggressive behaviour and in impulsive individuals with substance abuse disorder.

Results of RCTs are displayed in detail in Table 3. 

## 5. Efficacy of PUFAs on Impulsive Behaviours and Aggression in Attention Deficit Hyperactivity Disorder (ADHD)

Attention deficit hyperactivity disorder (ADHD) is a prevalent psychiatric disorder in the paediatric population [82] with onset before 7 years of age. The behavioural symptoms include inattention, impulsivity, hyperactivity, and often aggression. Patients with impulsive and aggressive behaviours in ADHD show a cortico-limbic dysfunction that underlies impulsive aggression and an aberrant prefrontal activity that is linked to poor response inhibition [83,84].

The primary treatment of ADHD involves pharmacotherapy with stimulant medications, such as methylphenidate, in combination with behavioural therapy. Although methylphenidate improves some conditions of comorbidity, its effectiveness in eliminating symptoms of ADHD is only partial [85]. Moreover, in children who display milder or subclinical levels of symptoms, an alternative treatment can be preferable to amphetamine-type stimulants. In this context has arisen the interest of researchers in nutritional supplementations.

Association between hyperactivity, impulsivity, and LC n-3 PUFA deficiency has been documented [86,87] and may be mediated by mechanisms that include insufficient dietary intake, lower rate of conversion of short-chain PUFAs to long-chain PUFAs, or rapid metabolism of long-chain PUFAs [88].

The neurobiological mechanism underlying the effect of LC n-3 PUFA supplementation in ADHD is not completely clear: some studies argue that neural integrity and function in prenatal development also depend on the levels of LC n-3 PUFAs. In particular, low levels of DHA in cord blood are associated with inattention and hyperactivity at 10 years of age [89].

Several trials have assessed the efficacy of PUFAs on oppositional behaviours, conduct problems, and aggression, which are core symptom dimensions in children or adolescents with ADHD.

Seventeen RCTs tested the effects of supplementation with PUFAs on the dimension of impulsive aggression in patients with ADHD aged between 4 and 18 years. Some studies have evaluated the efficacy of LC n-3 PUFAs (EPA and DHA), others have investigated the effects of a combination of LC n-3 and n-6 (gamma linolenic acid (GLA) and AA) PUFAs, and only one study compared the efficacy of LC n-3 PUFAs with that of LC n-6 PUFAs. Nine studies did not find significant correlation between PUFA treatment and amelioration of impulsivity and behavioural problems, while eight trials reported that supplementation of these agents significantly improved these symptom dimensions of the disorder. In these studies, EPA was administrated at doses ranging between 0.3 and 1.2 g/day, DHA between 0.3 and 0.65 g/day, GLA between 0.06 and 0.18 g/day, and AA at a dose of 0.04 g/day. Trials lasted 8 weeks to 12 months.

In order to assess impulsive and aggressive behaviours in ADHD, investigators used heterogeneous evaluation instruments, such as the Child Behaviour Checklist (CBCL) [90,91], Conners’ Parent Rating Scale (CPRS) and Teacher Rating Scale (CTRS) [92,93], and the Disruptive Behavior Disorders (DBD) Rating Scale [94]. In one study [95], aggression was assessed with a series of questions (for example, “Does he/she easily become angry/hostile?” or “Does he/she push, pat, kick other people or pull other people’s hair?”), while impulsivity was measured with the impatience test.

### 5.1. Efficacy of LC n-3 PUFAs in ADHD

Voigt et al. [90] provided in 54 ADHD children (42 males and 12 females) DHA or placebo for 4 months. All the patients received dietary supplementation in association with a maintenance therapy with stimulant medications. At the end of the trial, no changes of impulsivity were registered in this sample. In a similar way, a study performed by Hirayama et al. [95] treating 40 ADHD children (32 males and 8 females) with LC n-3 PUFAs alone or placebo did not register any changes in aggressive behaviours. Widenhorn-Müller et al. [91] evaluated in 95 children the effects of LC n-3 PUFAs (*n* = 46) against placebo (*n* = 49) on cognitive function and behavioural control. Supplementation with EPA and DHA improved working memory function but had no effect on other cognitive measures and behavioural control. In a study published by Anand and Sachdeva [96], clinical results did not reach statistical significance, although the 25 patients treated with LC n-3 PUFAs showed a greater reduction in all behavioural dimensions of ADHD compared with the 25 patients treated with placebo. 

Another RCT involving 40 boys with ADHD and 39 healthy controls compared the effect of the administration of LC n-3 PUFAs with that of placebo on behaviour and cognition [97]. Significant improvements in inattention scores were observed for both the ADHD and control groups, while no significant effects were observed on the CBCL (Child Behaviour Checklist) Rule Breaking and Aggressive Behavior subscales. In an RCT of 150 children performed by Manor et al. [93], the key finding was a significant score decrease in the Global-Restless/Impulsive subscale of Conners’ Parent and Teacher Rating Scales in the group treated with LC n-3 PUFAs. Furthermore, children that switched to LC n-3 PUFA treatment from placebo showed a significant reduction in both parent- and teacher-rated subscale scores.

In an RCT performed by Gustafsson et al. [92], the authors tested the effect of EPA alone versus that of placebo in 92 children with oppositional and impulsive behaviours that were characterized by a lower concentration of EPA and a higher concentration of LC n-6 PUFAs in serum and blood cells at baseline. Supplementation of EPA entailed a proportional reduction in n-6 PUFA concentrations in favour of n-3 PUFAs and was associated with clinical improvement after 15 weeks of treatment. In a more recent study of the efficacy of EPA versus that of placebo, Chang et al. [98] reported that the EPA group (*n* = 48) improved more than the placebo group (*n* = 44) in attention and vigilance but not in impulsive behavioural control.

### 5.2. Efficacy of LC n-3 and n-6 PUFAs in ADHD

LC n-3 PUFAs were compared with LC n-6 PUFAs in one trial conducted on 37 children with ADHD who were randomly assigned to two treatment arms. Behavioural abnormalities improved in both groups, so also the efficacy of n-6 PUFAs cannot be excluded on the basis of these data [99]. However, these findings are in contrast with those of other studies performed in ADHD [92] and other psychiatric disorders, which indicated the efficacy of LC n-3 but not of LC n-6 fatty acids. Considering these controversial and contrasting findings, some investigations were aimed at evaluating the effects of the combination of LC n-3 and n-6 PUFAs.

Seventy-five patients (64 boys and 11 girls) were included in a study that failed to demonstrate the benefits of PUFAs on impulsivity [100]. This double-blind randomized placebo-controlled trial suggested that for the whole group of children and adolescents, supplementation of EPA, DHA, GLA, and vitamin E for 3 months was not statistically superior to that of placebo in improving impulsive control. There was a subgroup (26% of all the subjects in the active group, 7% in the placebo group) who responded with a clinical improvement in ADHD core symptoms and also in impulsivity, but this finding did not reach the statistical significance. Among the most recent studies that did not support the efficacy of PUFAs in the treatment of ADHD, a randomized placebo-controlled clinical trial [101] including 76 male adolescents with ADHD failed to show positive effects of EPA plus DHA with a low dose of GLA on aggression and impulsivity, together with depression and anxiety. Assareh et al. [102] enrolled 40 Iranian children who received EPA, DHA, and a low dose of LC n-6 PUFAs in association with methylphenidate. At the end of the period of study, the scores of inattention, hyperactivity, and impulsivity were not significantly different between groups.

Among RCTs that suggested a beneficial effect of PUFA supplementation on conduct problems, Stevens et al. [94] enrolled 47 children (41 males and 6 females) who were randomized to receive either a mixed fish oil containing DHA, EPA, GLA, and AA or an olive oil. For the majority of the outcome measures, improvement was consistently better in the PUFA group than in the olive oil group, but the treatment difference was significant on only two measures: parent-rated conduct problems and teacher-rated attention symptoms. Moreover, in the PUFA supplementation group, a greater number of participants showed a decrease in oppositional and deviant behaviours. In a similar way, Richardson and Puri [103] enrolled 41 children with ADHD and specific learning difficulties who were randomly allocated to a group receiving EPA + DHA + GLA + AA supplementation or a placebo group. The authors obtained significantly lower scores for cognitive problems and general behavioural problems in the group treated with PUFAs. A positive response with a combination of LC n-3 and n-6 PUFAs on impulsivity was also observed in a study including 167 Australian children (128 boys, 39 girls) who were treated without stimulant medications for 30 weeks [104].

In a study conducted by Perera et al. [105], 94 children (69 males and 25 females) received for 6 months dietary supplementation of a combination of LC n-3 and n-6 PUFAs (*n* = 48) or placebo (*n* = 46), both in association with methylphenidate and standard behavioural interventions. Aggressiveness and impulsivity significantly decreased with an enhancement of children’s cooperation with teachers and parents in the treatment group.

Barragan et al. [106] enrolled 90 subjects who were randomized to a group receiving LC n-3 PUFAs plus LC n-6 PUFAs (*n* = 30) or a group receiving long-acting methylphenidate (*n* = 30) or a group receiving a combination of PUFAs and methylphenidate (*n* = 30) for 12 months. Although slightly less effective than methylphenidate, LC n-3/n-6 PUFAs were found to be effective and well-tolerated by children with ADHD. The combination of LC n-3/n-6 PUFAs with methylphenidate did not offer any significant benefit over methylphenidate alone, but permitted the use of lower doses of this stimulant. ADHD symptoms decreased in all the treatment arms, but the symptoms of hyperactivity–impulsivity showed a significantly better improvement in the group who received LC n-3/n-6 PUFAs associated with methylphenidate than in the group treated with LC n-3/n-6 PUFAs alone. Reduction of symptoms was slower in the arms treated with n-3/n-6 PUFAs than in the methylphenidate arm. Scale scores were stabilized after 8 weeks of treatment, suggesting long-term stabilization with n-3/n-6 PUFAs either alone or in combination.

Discrepancies in the findings of trials may be due to differences in sample selection and data analysis. We can conclude that supplementation of PUFAs in children with ADHD did produce a reduction in symptom severity, but this change was not statistically significant in some RCTs. Future studies should assess the most appropriate doses of LC n-3 PUFAs and follow up patients for longer periods. They should also clarify whether LC n-6 PUFAs can be usefully combined with LC n-3 PUFA compounds to increase results in this clinical population. The possibility that a more optimal PUFA intake during early years may decrease the prevalence of ADHD in children and adolescents should also be examined.

Results of RCTs are displayed in detail in Table 4. 

## 6. Reviews and Meta-Analyses

Evidence suggests that LC n-3 PUFAs play a positive role in the treatment of several mental disorders and have been shown to improve both mood and behaviour. However, there is little consensus on whether LC n-3 PUFAs are beneficial for improving impulsivity and aggression. In the current literature, seven reviews and one meta-analysis have evaluated the efficacy of PUFAs in these psychopathological domains.

The examination of reviews and meta-analyses is of particular interest in order to explore whether we can draw final conclusions from the whole pool of data collected from single trials.

The meta-analysis performed by Gajos and Beaver in 2016 [15] involved 7173 participants with and without neurodevelopmental/behavioural disorders and supported the relationship between LC n-3 PUFA intake and reduced aggression in both child and adult populations, both within intervention trials and observational studies. Moreover, LC n-3 PUFAs were observed to have a stronger effect in reducing violent and aggressive behaviours, rather than misconduct behaviours, indicating their potential role in dealing with more serious aggressive actions towards others. On the other hand, LC n-3 PUFAs seemed to be less effective on disobedient behaviours typically observed in younger populations [15].

Regarding the reviews, five studies [13,27,60,107,108] found encouraging results about the efficacy of LC n-3 PUFAs on aggression and impulsivity. On the contrary, two other reviews [109,110] highlighted methodological shortcomings or inconclusive evidence. In a review performed by Hallahan and Garland [13], the authors analysed a number of psychopathological phenomena and psychiatric disorders characterized by impulsivity and identified a significant correlation between deficits of LC n-3 PUFAs and higher impulsivity levels. Furthermore, these clinical conditions were ameliorated by the supplementation of LC n-3 PUFAs, with an improvement of impulse dyscontrol. In a following review by the same authors [107], patients characterized by high levels of impulsivity, hostility, and aggression were considered. Data showed an association, at either the epidemiological or clinical level, between decreased levels of LC n-3 PUFAs and higher levels of anger, impulsivity, and hostility. The correction of this deficiency with supplementation of PUFAs may result in clinical improvement. Bozzatello et al. [108] explored the impact of fatty acids on impulsive and aggressive behaviours in a review that analysed the effects of supplementation of these compounds in psychiatric disorders. Although results are encouraging and there is some evidence supporting the use of LC n-3 PUFAs in the treatment of conditions characterized by high levels of impulsivity and aggression, the authors conclude that this area of psychopathology needs further investigations. In a review of intervention studies focusing on the relationship between LC n-3 PUFAs and aggressive behaviour, Choy and Raine [60] found a small effect size of PUFA supplementation in reducing aggressive and antisocial conducts in both children and adults. The authors proposed the hypothesis that the efficacy of LC n-3 PUFA intake might be stronger in more impulsive forms of aggressive behaviour. Bozzatello et al. [27] studied the efficacy of LC n-3 PUFAs in psychiatric disorders with a transdiagnostic perspective, analysing multiple dimensions, including impulsivity and aggressiveness. The authors presented evidence of the beneficial effects of EPA and DHA supplementation on reducing levels of impulsivity, outbursts of anger, and overt aggression.

Hamazaki and Hamazaki [109] performed a review in which the majority of studies showed the aggression/hostility-controlling effects of fatty acids. However, because of different assessment methods used in these studies, it appears to be difficult to come to a clear conclusion about the role of PUFAs as modulators of aggression and hostility.

In a review published by Appleton et al. [110], results on the effects of LC n-3 PUFAs on aggression and hostility were inconclusive. Although some clinical studies have found associations between LC n-3 PUFAs and these dimensions, others have failed in this purpose. The authors suggested that inconclusive results may be due to the fact that LC n-3 PUFA supplementation produces its effect mainly in stressful situations. In addition, these compounds may reduce levels of aggression when they are expressed in vulnerable situations or in individuals with an innate tendency towards violent behaviour. Despite heterogeneous and inconsistent results, the authors highlighted the need for oncoming high-quality investigations about this issue.

Concerning tolerability, lack of severe adverse effects and general good tolerability are significant reasons to carefully consider the therapeutic potential of these agents. The most common side effects reported in clinical trials were nausea and a fishy aftertaste, but they were mild and rarely induced discontinuation [111]. Available data are insufficient to establish a tolerable daily intake of DHA and EPA in monotherapy or in combination, but supplementation of EPA and DHA up to 5 g/day is not dangerous for the general population [112]. In particular, EPA and DHA are generally recognized as safe and well tolerated at doses up to 5 g/day in terms of bleeding risk, as pointed out by Yokoyama et al. [113] and Tanaka et al. [114]. In addition, doses up to 5 g/day, consumed for a maximum period of 12–16 weeks, do not significantly affect glucose regulation in both healthy and diabetic subjects [115,116,117,118], do not increase infection risk by the activation of inappropriate inflammatory responses [119], and do not induce cardiovascular risk by the alteration of lipid metabolism [120]. In fact, combined intake of EPA and DHA at a dose of 2–6 g/day and intake of DHA at a dose of 2–4 g/die are responsible for an LDL concentration increase (3%) but do not affect cardiovascular risk. Intake of EPA at a maximum dose of 4 g/day does not induce significant changes in LDL plasma levels [121].

## Figures and Tables

**Table 1:** Randomized controlled trials and open trials of omega-3 PUFAs focused on impulsivity and aggression in patients at ultra-high risk (UHR) of psychosis and schizophrenia.

Study (Year) [Ref.]	Intervention Arm(s)	Comparison Arm(s)	Sample	Treatment Duration	Results
Légaré et al., 2007 [40]	EPA 0.4 g + DHA 0.2 g 3 times daily + 400 IUs vitamin E	no comparison arm	12 violent male inpatients with chronic schizophrenia	12 weeks	↓ agitation (*p* = 0.015), ↓ psychopathology (*p* < 0.01)
	DHA 0.36 g/day + EPA 0.54 g/day + antipsychotics	placebo + antipsychotics	50 patients with stable schizophrenia	12 weeks	↓ violence (*p* = 0.04), but no improvement in positive and negative symptoms (*p* = 0.86)
Amminger et al., 2013 [35]	EPA 0.7 g/day + DHA 0.48 g/day + vitamin E 0.0076 g	placebo	15 young individuals at UHR of psychosis	12 weeks	↑ impulse control (*p* = 0.03), ↑ level of functioning (*p* = 0.01)
Qiao et al., 2020 [42]	DHA 0.36 g/day + EPA 0.54 g/day + antipsychotics	placebo + antipsychotics	67 acute hospitalized patients with stable schizophrenia	8 weeks	no improvement in hostility (*p* = 0.884) or psychopathology (*p* = 0.165)

**Table 2:** Randomized controlled trials and open trials of omega-3 PUFAs focused on impulsivity and aggression in personality disorders.

Study (Year) [Ref.]	Intervention Arm(s)	Comparison Arm(s)	Sample	Treatment Duration	Results
Zanarini and Frankenburg, 2003 [51]	Ethyl-EPA 1 g/day (with no standard psychiatric therapies)	placebo	30 females with BPD	8 weeks	↓ aggression (*p* < 0.0001)
Amminger et al., 2013 [35]	EPA 0.7 g/day + DHA 0.48 g/day + vitamin E 0.0076 g	placebo	15 adolescents with BPD who also met criteria for UHR for psychosis	12 weeks	↓ impulsivity (*p* = 0.03)
Bellino et al., 2014 [55]	EPA 1.2 g/day + DHA 0.8 g/day + valproate 800–1300 mg/day	valproate 800–1300 mg/day	43 BPD outpatients	12 weeks	↓ impulsivity, anger, and self-mutilating conducts in the omega-3 fatty acid group (*p* ≤ 0.05)
Bozzatello et al., 2018 [56]	EPA 1.2 g/day + DHA 0.8 g/day + valproate 800–1300 mg/day	valproate 800–1300 mg/day	43 BPD outpatients	24 weeks’ follow-up	omega-3 fatty acid group showed long-lasting effects on anger control (*p* = 0.01)

**Table 3:** Randomized controlled trials and open trials of omega-3 PUFAs focused on impulsivity and aggression in impulse control and conduct disorders.

Study (Year) [Ref.]	Interventional Arm(s)	Comparison Arm(s)	Sample	Treatment Duration	Results
Gesh et al., 2002 [71]	EPA 0.08 g + DHA 0.044 g	placebo	172 young adult prisoners with externalizing disorder	from a minimum of 2 weeks to a maximum of 36 weeks	↓ violent offences (*p* = 0.03)
Raine at al., 2016 [72]	DHA 0.3 g + EPA 0.2 g	cognitive behavioural therapy (CBT) alone or PUFAs + CBT or placebo	290 children at high risk of conduct disorders	12 weeks + 24 weeks’ follow-up	The PUFAs-only group showed reduced externalizing behaviour compared with controls at 12 weeks (*p* = 0.044). The PUFAs + CBT group scored reduced externalizing behaviour compared with both the CBT-only (*p* = 0.031) and control groups (*p* = 0.023) at 24 weeks.
Raine at al., 2017 [73]	DHA 0.4112 g + EPA 0.604 g	social skills training or PUFAs + social skills training or placebo	282 children with externalizing behaviour	24 weeks + 24 weeks’ follow-up	Omega-3 fatty acid supplementation produced a long-lasting decline in child-reported reactive aggression (*p* = 0.042).
Buydens-Branchey et al., 2008 [79]	EPA + DHA at high dose (3 g/day)	placebo	24 patients with substance abuse	12 weeks	↓ outbursts of anger (*p* = 0.040)

**Table 4:** Randomized controlled trials and open trials of omega-3 PUFAs focused on impulsivity and aggression in patients with attention deficit hyperactivity disorder (ADHD).

Study (Year) [Ref.]	Interventional Arm(s)	Comparison Arm(s)	Sample	Treatment Duration	Results
Voigt et al., 2001 [90]	DHA 0.345 g/day + stimulant medication	placebo + stimulant medication	54 ADHD children 6–12 years old	4 months	no ↓ of impulsivity (*p* = 0.071) or of any ADHD symptom
Hirayama et al., 2004 [95]	DHA 3.6 g/week (2 cases under medication)	placebo (4 cases under medication)	40 ADHD children 6–12 years old	4 months	no ↓ aggressive behaviours (*p* = 0.053)
Johnson et al., 2009 [100]	EPA 0.558 g/day + DHA 0.174 g/day + GLA 0.06 g/day	placebo	75 ADHD children and adolescents 8–18 years old	6 months	no ↓ impulsive control (*p* = 0.08) or of other ADHD symptoms
Widenhorn- Müller et al., 2014 [91]	EPA 0.6 g/day + DHA 0.120 g/day	placebo	95 ADHD children 6–12 years old	16 weeks	no effects on behavioural symptoms (CBCL Aggressive Behavior, *p* = 0.29; Delinquent Behavior, *p* = 0.58) or other cognitive measures (LNS, *p* = 0.092; Digits Forward task, *p* = 0.46), but ↑ of working memory function (*p* = 0.019)
Anand and Sachdeva 2016 [96]	EPA 0.18 g and DHA+ atomoxetine 0.12 g	atomoxetine 0.5 mg/kg/day	50 ADHD children 4–11 years old	4 months	↓ hyperactive and impulsive symptoms, although the effect was not significant (*p* = 0.08)
Matsudaira et al., 2015 [101]	EPA 0.558 g and DHA 0.174 g, c-linoleic acid 0.06 g, and vitamin E 0.0096 g	placebo	76 male adolescents 12–16 years old	12 weeks	no improvement in aggression and impulsivity, depression and anxiety (*p* = 0.671)
Bos et al., 2015 [97]	EPA 0.65 g/day + DHA 0.65 g/day	placebo	40 young boys 8–14 years old with and without ADHD	16 weeks	↑ attention scores (*p* < 0.001), no significant effects on aggression
Assareh et al., 2017 [102]	DHA 0.241 g, EPA 0.033 g, and omega-6 fatty acids + methylphenidate 0.180 g	placebo + methylphenidate	40 ADHD patients 6–12 years old	10 weeks	no improvement in impulsivity (*p* = 0.919), inattention (*p* = 0.691), and hyperactivity (*p* = 0.811)
Chang et al., 2019 [98]	EPA 1.2 g/day	placebo (soybean oil 1.2 g)	92 ADHD children and adolescents 6–18 years old	12 weeks	↑ attention (*p* = 0.041), ↑ focused attention (*p* = 0.015), and ↑ vigilance (*p* = 0.036), but no improvement in impulsive behavioural control
Richardson and Puri, 2002 [103]	EPA 0.186 g/day + DHA 0.480 g/day + linolenic acid 0.864 g/day + arachidonic acid 0.04 g/day	placebo	41 children 8–12 years old with ADHD and specific learning difficulties	12 weeks	↓ cognitive problems (*p* = 0.01) and ↓ general behavioural problems (*p* = 0.02)
Stevens et al., 2003 [94]	DHA 0.48 g/day + EPA 0.08 g/day + arachidonic acid 0.04 g/day + GLA 0.096 g/day, vitamin E 0.024 g/day	placebo (olive oil 6.4 g/day)	50 children 6–13 years old with ADHD-like symptoms	4 months	↓ conduct problems (*p* = 0.05), ↑ attention symptoms (*p* = 0.03), ↓ oppositional and deviant behaviours (*p* = 0.02)
Sinn and Bryan, 2007 [104]	EPA 0.558 g + DHA 0.174 g + GLA 0.060 g + vitamin E 0.018 g	placebo (palm oil)	167 Australian children 7–12 years old	30 weeks	↓ impulsivity/hyperactivity (*p* < 0.01), inattention (*p* < 0.01)
Manor et al., 2012 [93]	PS 0.3 g and EPA 0.120 g + DHA (EPA/DHA ratio of 2:1)	placebo	150 children	15 weeks	↓ impulsivity (*p* < 0.05) and other ADHD symptoms
Bélanger et al., 2009 [99]	EPA 0.02–0.025 g/kg/day + DHA 0.85–0.105 g/kg/day	placebo (sunflower oil 500 mg)	37 children 6–11 years old with ADHD	16 weeks	↓ behavioural problems and inattention (*p* < 0.05)
Gustafsson et al., 2010 [92]	EPA 0.5 g/day	placebo	92 ADHD children 7–12 years old	15 weeks	↓ symptoms in two ADHD subgroups: oppositional (*p* = 0.01) and less hyperactive/impulsive children (*p* = 0.03)
Perera et al., 2012 [105]	omega-3 fatty acids 0.29637 g + omega-6 0.18075 g (ratio of 1.6:1) + methylphenidate and standard behavioural intervention	placebo with ADHD medications	98 ADHD children 6–12 years old	6 months	↓ aggressiveness (*p* = 0.000), ↓ impulsiveness (*p* = 0.009) and ↑ cooperation with teachers and parents (*p* = 0.009), no improvement in distractibility
Barragan et al., 2017 [106]	omega-3/6, fatty acids, or combination of omega-3/6 fatty acids and methylphenidate	methylphenidate	90 ADHD children 6–12 years old	12 months	↓ hyperactivity–impulsivity in the group treated with omega-3/6 + MPH vs. omega-3/6 alone (*p* = 0.009), no significant difference in hyperactivity–impulsivity between omega 3/6 treatment and MPH alone, and ↓ adverse events in the group with combined treatment vs. MPH alone (*p* = 0.001)

## Abbreviations

PUFAs	Polyunsaturated fatty acids
EPA	Eicosapentaenoic acid
DHA	Docosahexaenoic acid
ethyl-EPA	ethyl-eicosapentaenoic acid
GLA	Gamma-linolenic acid
PS	phosphatidylserine
↓	Decrease of
↑	Increase of
IUs	International Units
>	Superior of
<	Inferior of
ADHD	Attention Deficit Hyperactivity Disorder
BPD	Borderline Personality Disorder
LNS	Letter-Number Sequencing
